# Neuroonkologie: Herausforderungen und Perspektiven

**DOI:** 10.1007/s00115-023-01603-3

**Published:** 2024-02-07

**Authors:** Ghazaleh Tabatabai, Uwe Schlegel

**Affiliations:** 1 Abteilung Neurologie mit interdisziplinärem Schwerpunkt Neuroonkologie, Neurologische Universitätsklinik, Universitätsklinikum Tübingen, Hoppe-Selyer-Str. 3, 72074 Tübingen, Deutschland; 2 Hertie-Institut für Klinische Hirnforschung, Eberhard Karls Universität Tübingen, Tübingen, Deutschland; 3 Neurologische Klinik, Neuroonkologischer Schwerpunkt, Klinik Hirslanden, Witellikerstr. 40, 8032 Zürich, Schweiz

Erkenntnisgewinne in der klinischen Neurologie und in den translational orientierten Neurowissenschaften sind sehr dynamisch und vollziehen sich so rasant, dass es Nichtspezialisten mitunter schwerfällt, auf dem Laufenden zu bleiben. Dies gilt auch für das Gebiet der Neuroonkologie. Neuroonkologische Erkrankungen sind Erkrankungen des Nervensystems. Die umfassende Betreuung betroffener Patientinnen und Patienten erfordert eine interdisziplinäre Zusammenarbeit in einem Expertenteam. Aber gerade Neurologinnen und Neurologen aus dem deutschsprachigen Raum haben in der letzten Dekade international sichtbare Beiträge geleistet und Impulse gegeben, die zu vertieftem tumorbiologischem Verständnis und zu potenziellen neuen therapeutischen Strategien führen.

International sichtbare und wegweisende Impulse aus dem deutschsprachigen Raum

Mit der vorliegenden Ausgabe wollen wir die Leserinnen und Leser von *Der Nervenarzt* auf eine faszinierende Reise durch aktuelle Gebiete aus klinischer und neuroonkologischer Grundlagenforschung nehmen. Die therapeutischen Implikationen einiger dieser Einblicke sind zwar (noch) nicht konkret absehbar. Aus unserer Sicht ist eine Würdigung dieser Erkenntnisse für die geschätzte Leserschaft von *Der Nervenarzt* dennoch bereits jetzt ein Gewinn, weil es sich um bahnbrechende Befunde handelt, die unser biologisches Verständnis von Tumoren im Nervensystem revolutionieren und neue Strategien zielgerichteter immunologischer und zellbasierter Therapien eröffnen könnten. Zudem widmen wir uns einer fortgesetzten Erfolgsgeschichte der systemischen Tumortherapie bei Primären ZNS-Lymphomen und beleuchten im abschließenden Artikel ein sicher bis dato unterschätztes, zumindest therapeutisch oft ungenügend adressiertes Problem, die behandlungsbedürftige Depression zahlreicher Patienten mit malignen Tumoren im Nervensystem.

Der Artikel von *Frank Winkler* und *Varun Venkatamarani* beleuchtet eine Entdeckung der Arbeitsgruppe von Frank Winkler am Deutschen Krebsforschungszentrum Heidelberg und der dortigen Neurologischen Universitätsklinik, die 2015 in der Zeitschrift *Nature* publiziert wurde. Dabei war der Befund, dass maligne (astrozytäre, nichtoligodendrogliale) Gliome lange Fortsätze („tumor microtubes“) ausbilden, darüber mit ihren Schwesterzellen kommunizieren und abgetötete Gliomzellen mit Nuklei aus einer Mitose distanter Tumorzellen „revitalisieren“ können, nur der Beginn einer faszinierenden, dabei erschreckenden Entdeckung der Biologie maligner Gliome, die in der Folgezeit weiter entschlüsselt wurde: So können Gliomzellen über eben diese „microtubes“ an Neuronen andocken und mit diesen funktionelle Synapsen bilden, neuronale Erregung bahnen und umgekehrt durch parakrine und autokrine Mechanismen nach dieser neuronalen Erregung Impulse zur Zellproliferation und zum Tumorwachstum nutzen. Dass maligne Gliome zudem intrinsische neuronale (embryonale) Programme nutzen, um sich zu vernetzen, um Proliferation und Invasion zu propagieren und um Therapieresistenz zu lernen, ist das neueste Kapitel dieser Forschungsgeschichte. Wie man dies nun therapeutisch nutzen kann, um mithilfe von Rezeptoren- und Ionenkanalblockern das funktionale Netzwerk lahmzulegen, um möglicherweise aus einem aggressiven Tumor einen „schlafenden Mitbewohner“ zu machen, erläutern *Varun Venkatamarani *und* Frank Winkler* in ihrem Artikel.

*Lisa Sevenich *und* Dieter Hendrik Heiland* beleuchten in ihrem Artikel jene biologischen Vorgänge in der Tumorumgebung, die bislang in der Therapiekonzeption eher vernachlässigt worden sind. Tumorzellen bereiten sich durch eine Vielzahl zunehmend entschlüsselter molekularer Mechanismen eine Art „Wohlfühlnische“ im Nervensystem. Das Ziel dieser Nischenbildung, um die Tumoren sogar eigene Barrikaden aufbauen, ist es z. B., verborgen vor dem Immunsystem unbehelligt wachsen zu können. Ein vertieftes Verständnis dieser Tumorumgebung und die Identifikation zellulärer Akteure, die im orchestrierten Zusammenspiel den Tumorzellen dabei helfen, sich im Nervensystem einzunisten, eröffnen bis dato ungenutzte neue therapeutische Potenziale.

Die Vision einer effektiven Mobilisierung des Immunsystems im Kampf gegen Tumoren im Nervensystem wird in der Neuroonkologie schon seit Dekaden verfolgt. Zahlreiche Rückschläge haben nicht dazu geführt, diese Mission zu stoppen. Informationen aus gescheiterten Ansätzen bildeten im Gegenteil die Grundlagen der Weiterentwicklung dieser Mission in aktuellen translational orientierten Forschungsprojekten und Phase-1-Studien. *Katharina Sahm *und* Tobias Weiss *geben in ihrem Artikel einen konzisen Überblick über aktuelle Strategien und legen auch sehr eindrücklich dar, warum es wichtig ist, Immuntherapien weiter konsequent als experimentelle Ansätze in der Neuroonkologie zu verfolgen. Unverändert sind Immuntherapien experimentelle Verfahren, die in klinischen Therapiestudien an erfahrenen Zentren zu evaluieren sind – und somit aus Sicht der Herausgeber dieser Ausgabe ungeeignet für kommerzielle Behandlungsangebote.

Primäre ZNS-Lymphome sind selten, hochaggressiv und unbehandelt innerhalb von wenigen Wochen oft tödlich. Dass man sie effektiv (ohne Operation und Strahlentherapie) mit hochdosiertem Methotrexat behandeln kann, haben internistische Onkologen entdeckt. In der Onkologie, aber auch in der Neurologie wurden dann komplexe Chemotherapieprotokolle entwickelt und in Studien eingesetzt, die zur Heilung einer Untergruppe von Patienten führten. Gerade für die Patienten mit ungenügendem Therapieansprechen sind derzeit neue zielgerichtete Therapien in klinischer Erprobung, zu denen auch die „lebenden Medikamente“ CAR-T-Zellen, also zytotoxische, individuelle Patienten-T-Zellen mit einem chimären, speziell für diese Lymphome genetisch konstruierten Antigenrezeptor gehören**. ***Louisa von Baumgarten*, die in Deutschland zu den Pionierinnen dieser Methode zählt, *Leon Kaulen *und* Sabine Seidel* fassen in ihrer Übersicht die aktuellen Therapieentwicklungen zusammen.

Lebensqualität ist ebenso entscheidend für Betroffene mit lebensbegrenzenden Tumorerkrankungen wie Lebenszeit. Mitunter erzählen Betroffene nicht von ihrer Verzweiflung und depressiven Herabstimmung, oft fragen wir Behandler noch nicht einmal, vielleicht weil wir fürchten, keine angemessene Antwort auf die existenziellen Fragen unseres Gegenübers geben zu können. Aber wir berauben uns mit der Nichteinleitung einer wirksamen antidepressiven medikamentösen Therapie einer Behandlungsmöglichkeit, die Lebensqualität entscheidend verbessern kann. Hier sollten wir Neurologen deutlich mehr Initiative ergreifen und im Übrigen die Behandlung reaktiver psychiatrischer Erkrankungen bei unseren Patienten nur in Ausnahmefällen an unsere Kollegen in der Psychiatrie delegieren bzw. nicht „einfach“ mitbetreuenden Psychoonkologen überlassen. Wie oft wir Neurologen hier zu kurzsichtig sind und viel zu kurz zielen und wie wir dies verbessern können, zeigen uns *Corinna Seliger-Behme, Thomas Kowalski *und* Johannes Knabbe* im abschließenden Artikel dieser Ausgabe.

Wir wünschen allen Leserinnen und Lesern viel Freude beim Lesen.

Mit herzlichen Grüßen

Ihre

Ghazaleh Tabatabai und Uwe Schlegel

